# Analysis of volume and topography of adipose tissue in the trunk: Results of MRI of 11,141 participants in the German National Cohort

**DOI:** 10.1126/sciadv.add0433

**Published:** 2023-05-12

**Authors:** Tobias Haueise, Fritz Schick, Norbert Stefan, Christopher L. Schlett, Jakob B. Weiss, Johanna Nattenmüller, Katharina Göbel-Guéniot, Tobias Norajitra, Tobias Nonnenmacher, Hans-Ulrich Kauczor, Klaus H. Maier-Hein, Thoralf Niendorf, Tobias Pischon, Karl-Heinz Jöckel, Lale Umutlu, Annette Peters, Susanne Rospleszcz, Thomas Kröncke, Norbert Hosten, Henry Völzke, Lilian Krist, Stefan N. Willich, Fabian Bamberg, Juergen Machann

**Affiliations:** ^1^ Institute for Diabetes Research and Metabolic Diseases, Helmholtz Center Munich at the University of Tuebingen, Tuebingen, Germany.; ^2^ German Center for Diabetes Research (DZD), Tuebingen, Germany.; ^3^ Section on Experimental Radiology, Department of Diagnostic and Interventional Radiology, University Hospital Tuebingen, Tuebingen, Germany.; ^4^ Department of Internal Medicine, Division of Diabetology, Endocrinology and Nephrology, Eberhard-Karls University Tuebingen, Tuebingen, Germany.; ^5^ Department of Diagnostic and Interventional Radiology, Medical Center–University of Freiburg, Faculty of Medicine, University of Freiburg, Freiburg, Germany.; ^6^ Department of Diagnostic and Interventional Radiology, University Hospital Heidelberg, Heidelberg, Germany.; ^7^ Division of Medical and Biological Informatics, German Cancer Research Center, Heidelberg, Germany.; ^8^ Division of Medical Image Computing, German Cancer Research Center, Heidelberg, Germany.; ^9^ Pattern Analysis and Learning Group, Department of Radiation Oncology, Heidelberg University Hospital, Heidelberg, Germany.; ^10^ Berlin Ultrahigh Field Facility (B.U.F.F.), Max-Delbrueck Center for Molecular Medicine in the Helmholtz Association, Berlin, Germany.; ^11^ Experimental and Clinical Research Center, A Joint Cooperation Between the Charité Medical Faculty and the Max-Delbrueck Center for Molecular Medicine in the Helmholtz Association, Berlin, Germany.; ^12^ Max-Delbrueck-Center for Molecular Medicine in the Helmholtz Association (MDC), Molecular Epidemiology Research Group, Berlin, Germany.; ^13^ Max-Delbrueck-Center for Molecular Medicine in the Helmholtz Association (MDC), Biobank Technology Platform, Berlin, Germany.; ^14^ Berlin Institute of Health at Charité–Universitätsmedizin Berlin, Core Facility Biobank, Berlin, Germany.; ^15^ Charité–Universitätsmedizin Berlin, corporate member of Freie Universität Berlin and Humboldt-Universität zu Berlin, Berlin, Germany.; ^16^ Institute for Medical Informatics, Biometry and Epidemiology (IMIBE), University Hospital Essen, Essen, Germany.; ^17^ Department of Diagnostic and Interventional Radiology and Neuroradiology, University Hospital Essen, Essen, Germany.; ^18^ Department of Epidemiology, Institute for Medical Information Processing, Biometry and Epidemiology, Ludwig-Maximilians-Universität München, Munich, Germany.; ^19^ Institute of Epidemiology, Helmholtz Center Munich, German Research Center for Environmental Health, Neuherberg, Germany.; ^20^ German Center for Cardiovascular Research (DZHK), Partner Site Munich Heart Alliance, Munich, Germany.; ^21^ German Center for Diabetes Research (DZD), Partner Site Neuherberg, Neuherberg, Germany.; ^22^ Department of Diagnostic and Interventional Radiology, University Hospital Augsburg, Faculty of Medicine, University of Augsburg, Augsburg, Germany.; ^23^ Centre for Advanced Analytics and Predictive Sciences (CAAPS), University Augsburg, Augsburg, Germany.; ^24^ Institute of Diagnostic Radiology and Neuroradiology, University Medicine Greifswald, Greifswald, Germany.; ^25^ Institute for Community Medicine, University Medicine Greifswald, Greifswald, Germany.; ^26^ German Centre for Cardiovascular Research (DZHK), Partner Site Greifswald, Greifswald, Germany.; ^27^ Institute of Social Medicine, Epidemiology and Health Economics, Charité–Universitätsmedizin Berlin, Berlin, Germany.

## Abstract

This research addresses the assessment of adipose tissue (AT) and spatial distribution of visceral (VAT) and subcutaneous fat (SAT) in the trunk from standardized magnetic resonance imaging at 3 T, thereby demonstrating the feasibility of deep learning (DL)–based image segmentation in a large population-based cohort in Germany (five sites). Volume and distribution of AT play an essential role in the pathogenesis of insulin resistance, a risk factor of developing metabolic/cardiovascular diseases. Cross-validated training of the DL-segmentation model led to a mean Dice similarity coefficient of >0.94, corresponding to a mean absolute volume deviation of about 22 ml. SAT is significantly increased in women compared to men, whereas VAT is increased in males. Spatial distribution shows age- and body mass index–related displacements. DL-based image segmentation provides robust and fast quantification of AT (≈15 s per dataset versus 3 to 4 hours for manual processing) and assessment of its spatial distribution from magnetic resonance images in large cohort studies.

## Teaser

Automated analysis of body fat at population level provides insight into anthropometric associations with metabolic risk.

## INTRODUCTION

The obesity pandemic is growing rapidly; in 2016, 39% of the adult world population was overweight, and 13% were obese. The worldwide prevalence has nearly tripled since 1975 (*1*). Abdominal obesity, as manifested by increased visceral adipose tissue (VAT) (*2*), shows a strong correlation to insulin resistance and is a key condition of the metabolic syndrome, which is associated with the risk of developing type 2 diabetes (*3*–*5*) and a major risk factor for a wide range of other diseases (*6*, *7*) such as cardiovascular diseases (*8*, *9*) and several types of cancers (*10*, *11*).

Not only the volume of adipose tissue (AT) but also its regional distribution are considered to play an essential role in the pathogenesis of insulin resistance (*12*, *13*), implying the necessity to characterize individuals for body fat distribution in addition to exclusively determine simple anthropometric measures as, e.g., body mass index (BMI) or waist-to-hip ratio, as, especially VAT shows a better correlation to metabolic parameters (*9*, *14*–*17*). For example, regarding metabolically healthy obesity, in the Tübingen Diabetes Family Study (TDFS), the metabolically healthy and insulin-sensitive obese individuals were found to differ in liver fat content, intramyocellular lipids, and VAT but not in body weight, height, or waist circumference (WC), from the metabolically unhealthy and insulin-resistant obese individuals (*14*). Furthermore, in the TDFS, insulin secretion failure, insulin resistance, fatty liver [measured by ^1^H magnetic resonance (MR) spectroscopy], and MR imaging (MRI)–determined visceral obesity, but not BMI categories or visceral obesity based on WC measurement, were independent determinants of prediabetes (*18*). Therefore, noninvasive assessment using whole-body MRI, which is able to precisely distinguish between VAT and subcutaneous adipose tissue (SAT), has been established (*19*) and can be regarded as gold standard for the assessment of topography and quantification of AT. State-of-the-art MRI techniques enable gapless acquisitions with high spatial resolution as provided by three-dimensional (3D) chemical shift selective MRI using Dixon-based techniques (*20*, *21*). Large population-based cohort studies such as the German National Cohort (GNC) (*22*) or the U.K. Biobank (*23*) provide comprehensive databases for the assessment of AT depots from MRI (*24*).

Volumetric localization and quantification of AT from MRI are based on slice-wise semantic segmentation of AT compartments. Manual segmentation requires trained personnel, is time-consuming, is costly and—especially in large cohort studies using whole-body images—not feasible in practice. Recent studies have implemented automated segmentation algorithms using atlas-based segmentation (*21*, *25*, *26*), statistical shape models (*27*, *28*), or machine learning (*29*–*31*) on 2D or 3D data using 2D and 3D segmentation algorithms (*32*) and demonstrated the applicability of the methods in small- to medium-sized populations.

Because of the success of deep learning (DL) algorithms in medical image analysis (*33*), many task-specific and highly specialized DL models, often mainly focused on improving model training evaluation metrics, have been proposed (*30*, *31*, *34*, *35*). Because of specific assumptions on input data and nontrivial, often undocumented configuration, the applicability of most of these models in a broader scientific setting is limited. Furthermore, these models do not necessarily output anatomically accurate results despite improved training evaluation metrics, as time resources are invested in an iterative trial-and-error process during method design instead of providing accurate examples of manual segmentation (model-centric DL). Recently, this issue was addressed by the introduction of nnU-Net (*36*). This framework quantitatively confirms that the configuration (including data processing pipeline, training parameters, etc.) of a DL model has more impact on its performance than architectural variations. Consequently, nnU-Net enables cross-task generalization and can be used as an out-of-the-box tool (*36*) paving the way toward data-centric artificial intelligence that is focused on applications of DL by improving its underlying data (*37*).

The purpose of this study is to assess the volume of different AT compartments of the body trunk, i.e., VAT ranging from hip to cardiac apex, and SAT, which is differentiated in subcutaneous abdominal adipose tissue (SAAT) and subcutaneous thoracic adipose tissue (STAT) using the cardiac apex as the boundary and their spatial distribution along the craniocaudal axis, thereby demonstrating the feasibility of using DL-based image segmentation in a large population-based cohort undergoing MRI.

## RESULTS

### Automatic data processing using DL segmentation model

**Fig. 1.**
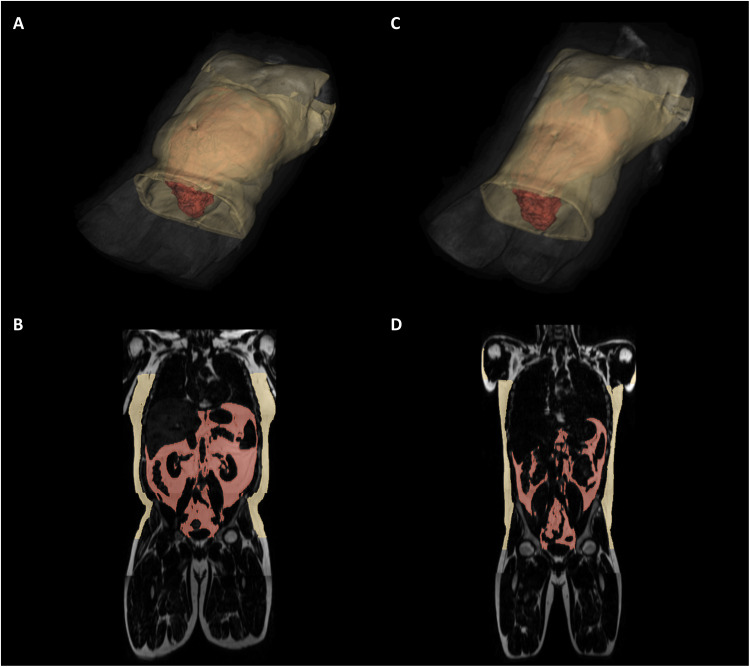
Examples of successful segmentations. 3D view and exemplary coronal slice of segmented SAT (yellow) and VAT (red). (**A** and **B**) Male participant, 42 years; BMI, 34.5 kg/m^2^; SAAT, 10.5 liters; STAT, 4.2 liters; VAT, 6.8 liters. (**C** and **D**) Male participant, 26 years; BMI, 22.9 kg/m^2^; SAAT, 5.9 liters; STAT, 2.8 liters; VAT, 2.4 liters.

**Fig. 2.**
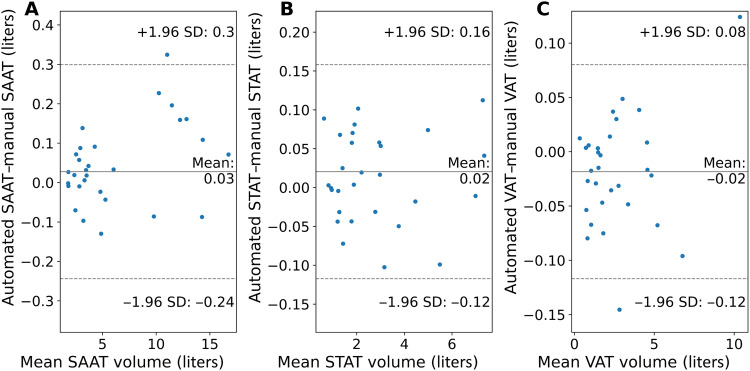
Agreement of manual and automated segmentation. Bland-Altman plots showing the agreement of SAAT (**A**), STAT (**B**), and VAT (**C**) quantification.

On the basis of 30 stratified randomly selected samples from the GNC, fivefold cross-validated training of the nnU-Net segmentation model (exemplary results shown in Fig. 1) led to mean Dice similarity coefficients (DSCs) for VAT, SAAT, and STAT of 0.947 ± 0.033 (0.855 to 0.983), 0.981 ± 0.011 (0.933 to 0.993), and 0.955 ± 0.028 (0.850 to 0.984), corresponding to a mean absolute volume deviation of AT volume of −18.4, 27.5, and 20.3 ml, respectively. Bland-Altman plots (see Fig. 2) showed good agreement and low bias of manual and automated quantification of AT in all three compartments. Comprehensive cross-validation model performance metrics are summarized in Table 1. Intrareader similarity (IRS) of the main annotator is 0.916 (SAAT), 0.876 (STAT), and 0.777 (VAT).

**Table 1.** Model performance metrics. Mean validation metrics of the class-wise confusion matrix and SD of the fivefold cross-validated model training using a total of 30 annotated datasets.

	SAAT	STAT	VAT
Accuracy	0.998 ± 0.001	0.995 ± 0.002	0.998 ± 0.001
Dice	0.981 ± 0.011	0.955 ± 0.028	0.947 ± 0.033
False discovery rate	0.022 ± 0.014	0.050 ± 0.037	0.047 ± 0.032
False-negative rate	0.017 ± 0.014	0.040 ± 0.027	0.058 ± 0.038
False-omission rate	0.001 ± 0.001	0.002 ± 0.001	0.001 ± 0.001
False-positive rate	0.001 ± 0.001	0.003 ± 0.002	0.001 ± 0.001
Jaccard	0.962 ± 0.027	0.915 ± 0.048	0.902 ± 0.057
Negative predictive value	0.999 ± 0.001	0.998 ± 0.001	0.999 ± 0.001
Precision	0.979 ± 0.015	0.950 ± 0.037	0.953 ± 0.032
Recall	0.983 ± 0.014	0.960 ± 0.027	0.942 ± 0.038
True-negative rate	0.999 ± 0.001	0.997 ± 0.002	0.999 ± 0.001
Relative error (%)	0.52 ± 1.87	1.17 ± 3.94	−1.25 ± 2.79
Absolute error (ml)	27.5 ± 140.9	20.3 ± 71.5	−18.4 ± 51.1

**Fig. 3.**
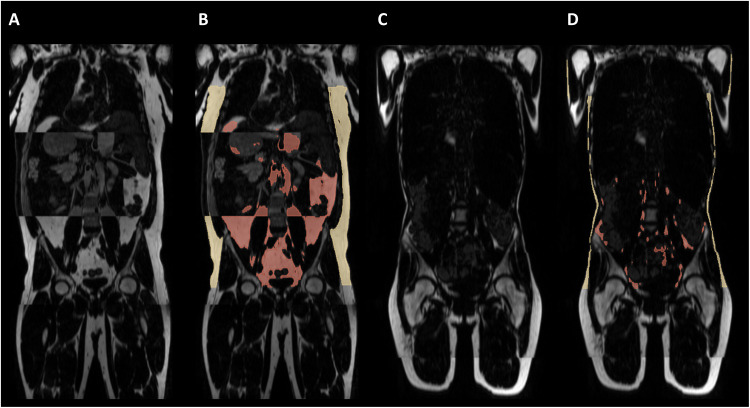
Outlier detection. Examples of detected outliers based on model uncertainty. (**A** and **B**) True-positive detected outlier due to partial fat-water swap in the abdomen; quantification of AT is not possible [(A) without model–generated segmentation and (B) model-generated segmentation]. (**C** and **D**) False-positive detected outlier due to low AT volume [(C) without model–generated segmentation and (D) model-generated segmentation]; quantification of AT is possible.

On the basis of a population of 11,191 participants of the GNC, the application of the trained segmentation model led to the uncertainty-based detection of 217 (about 2% of the entire population) potential outliers. After their manual inspection, 21 participants (about 10% of the automatically initially classified outliers) had to be excluded because of imaging errors (partial fat-water swaps) (see Fig. 3, A and B) in the abdomen. False-positive outliers that could be kept after manual inspection mostly include participants with very low AT volume (see Fig. 3, C and D). Manual inspection of 1120 additional participants was unremarkable. In addition, two participants had to be excluded because of corrupted image data, and 27 had to be excluded because of missing height or weight measurements yielding a total of 11,141 participants (5708 males and 5433 females) for AT quantification to form the study population. WC was available from 11,117 participants (5697 males and 5420 females). Anthropometric data of the analyzed study population can be found in Table 2.

**Table 2.** Study population. Anthropometric data and AT volumes obtained from automatic segmentation of the entire study population and anthropometrics of the training data. *P < 0.05.

		Male		Female	
		Means ± SD	Range	Means ± SD	Range
Study population	*n*	5708	–	5433	–
	Age (years)	52.1 ± 11.4	20–72	51.7 ± 11.3	20–72
	Height (kg)	178.2 ± 7.0	152.8–204.6	164.9 ± 6.5	126.4–189.0
	Weight (kg)	86.9 ± 14.3	45.8–191.6	71.2 ± 14.4	37.9–157.5
	BMI (kg/m^2^)	27.4 ± 4.1	15.6–49.4	26.2 ± 5.2	16.2–54.6
	WC† (cm)	97.4 ± 12.1	63.2–165.0	86.1 ± 13.1	54.0–150.0
	SAAT (liters)	6.17 ± 3.05	0.66–26.3	7.68 ± 3.88	0.83–29.4
	STAT (liters)	2.81 ± 1.17	0.36–13.6	3.78 ± 1.77	0.38–16.0
	VAT (liters)	4.84 ± 2.36	0.54–15.3	2.51 ± 1.55	0.22–10.3
Training data‡	*n*	15	–	15	–
	Age (years)	44.5 ± 14.7*	24–69	44.9 ± 13.4*	23–64
	Height (kg)	175.5 ± 8.0	164.4–190.6	164.5 ± 8.2	152.6–176.5
	Weight (kg)	81.0 ± 15.3	58.7–111.9	66.1 ± 20.0	46.7–106.4
	BMI (kg/m^2^)	26.6 ± 6.4	20.0–37.5	24.6 ± 7.9	18.2–37.0
	WC (cm)	91.9 ± 14.6	72.7–125.0	82.0 ± 17.5	64.1–111.6

†Analysis included all individuals with WC data available (*n* = 5697 males and *n* = 5420 females).

‡Differences between the complete study population and the training subset are tested for significance.

### Assessment of AT volume and distribution

**Fig. 4.**
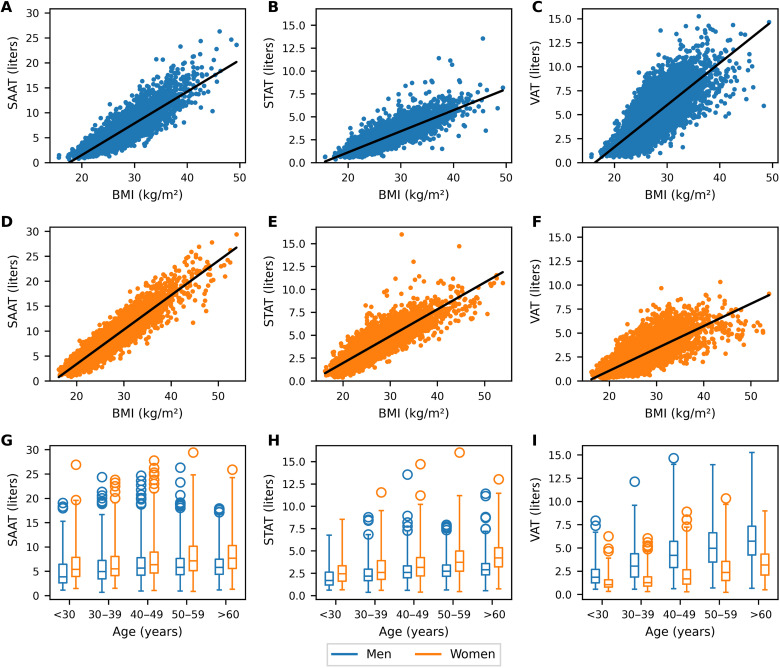
Anthropometric associations. Association and linear regression of AT compartments with BMI [SAAT for males (**A**) and females (**D**), STAT for males (**B**) and females (**E**), and VAT for males (**C**) and females (**F**)] and with age [SAAT (**G**), STAT (**H**), and VAT (**I**)].

Regarding the entire study population, females were characterized by significantly higher SAAT and STAT compared to males (7.68 ± 3.88 and 3.78 ± 1.77 liters for females and 6.17 ± 3.05 and 2.81 ± 1.17 liters for males, respectively; see Fig. 4, first and second column, A, B, D, E, G, and H). Males had significantly higher VAT volume (4.84 ± 2.36 liters for males and 2.51 ± 1.55 liters for females; see Fig. 4, last column, C, F, and I).

Females showed a stronger correlation of SAAT, STAT, and VAT with BMI compared to males. SAAT showed the strongest correlation with BMI in both genders. All correlation coefficients are summarized in Table 3. Moreover, normal-weight individuals of both genders show variability in VAT (0.5 to 9.4 liters for males and 0.2 to 5.9 liters for females) and SAAT (0.7 to 8.9 liters for males and 0.8 to 11.3 liters for females). The range of variability of VAT (1.6 to 15.3 liters for males and 1.2 to 10.3 liters for females) and SAAT (3.4 to 26.3 liters for males and 5.9 to 29.4 liters for females) is even greater for obese individuals indicating the presence of the “thin outside fat inside” phenotype with a high share of VAT despite being lean (*38*) and metabolically healthy obese individuals with a low share of VAT, despite being obese (*14*).

**Table 3.** Correlations with anthropometric data. *P < 0.05 and **P < 0.001.

	SAAT		STAT		VAT	
	Male	Female	Male	Female	Male	Female
Age	0.07^**^	0.15^**^	0.22^**^	0.28^**^	0.40^**^	0.41^**^
Height	0.10^**^	−0.03^*^	0.05^**^	−0.10^**^	−0.01	−0.11^**^
Weight	0.85^**^	0.91^**^	0.78^**^	0.82^**^	0.71^**^	0.74^**^
BMI	0.86^**^	0.93^**^	0.81^**^	0.87^**^	0.77^**^	0.79^**^
WC†	0.85^**^	0.89^**^	0.83^**^	0.86^**^	0.85^**^	0.84^**^

†Analysis included all individuals with WC data available (*n* = 5697 males and *n* = 5420 females).

Regarding the association with age, SAAT and STAT showed negligible correlation in both genders. VAT showed a moderate but significant positive correlation with age in both genders (see Table 3). Considering age decades, participants in the oldest group of the study population (age > 60 years) had 3.33 ± 1.60 liters of VAT compared to 1.27 ± 0.75 liters (+162%) in the youngest age group (age < 30 years) for women and 5.84 ± 2.34 liters of VAT compared to 2.18 ± 1.30 liters (+167%) for men with each age group showing wide variability (see Fig. 4, G to I).

Using anthropometric measures routinely collected in clinical practice (i.e., age, height, and weight) to explain the variation in MRI-assessed AT compartments showed that the addition of WC lead to an improved prediction for all AT compartments. While VAT showed no gender-specific differences (*R*^2^ = 0.75 for men and women), the variation of both subcutaneous AT compartments was better explained in women (*R*^2^ = 0.90 for SAAT and *R*^2^ = 0.80 for STAT) compared to men (*R*^2^ = 0.81 for SAAT and *R*^2^ = 0.72 for STAT). An overview of all models is provided in table S1.

**Fig. 5.**
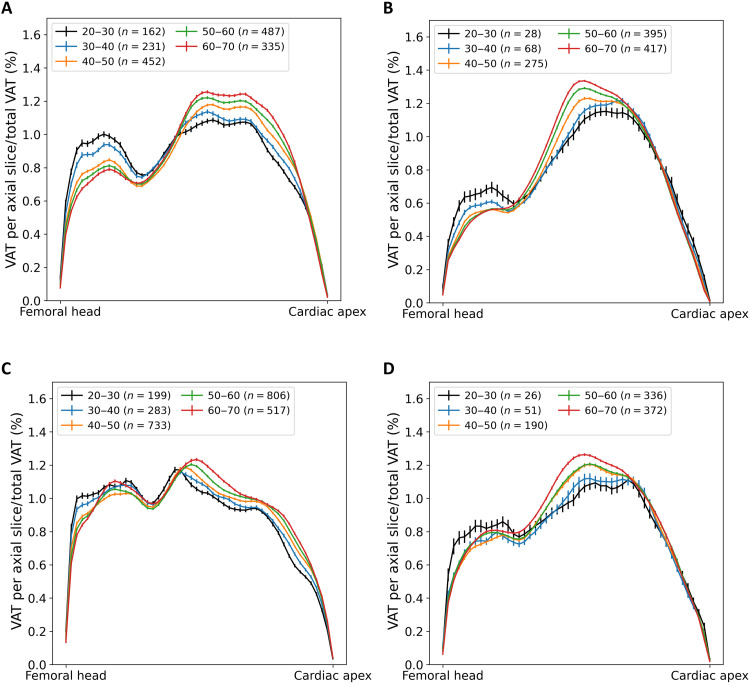
Spatial distribution of VAT. Age dependency of the regional distribution of VAT along craniocaudal axis. (**A**) Males with a BMI of <25.0 kg/m^2^, (**B**) males with a BMI of >30.0 kg/m^2^, (**C**) females with a BMI of <25.0 kg/m^2^, and (**D**) females with a BMI of >30.0 kg/m^2^. Error bars show SEM.

**Fig. 6.**
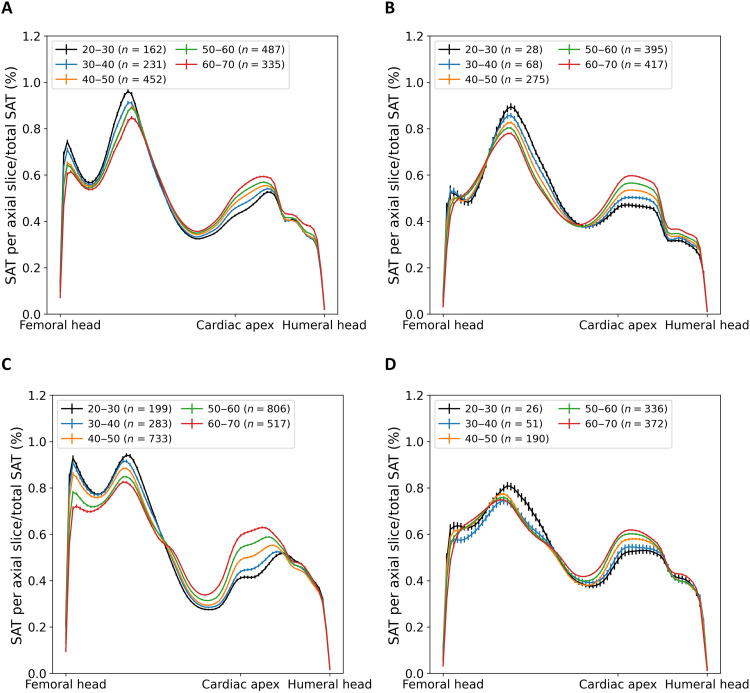
Spatial distribution of SAT. Age dependency of the regional distribution of SAT along craniocaudal axis. (**A**) Males with a BMI of <25.0 kg/m^2^, (**B**) males with a BMI of >30.0 kg/m^2^, (**C**) females with a BMI of <25.0 kg/m^2^, and (**D**) females with a BMI of > 30.0 kg/m^2^]. Error bars show SEM.

Regarding the regional spatial distribution of VAT along the craniocaudal axis, there were significant age-dependent differences in the group of normal-weight males. VAT shifts from the pelvis to the abdomen with increasing age. Normal-weight females do not show such displacement (see Fig. 5, A and C). In addition, obese males had less VAT in the pelvis region and showed an age-dependent displacement of VAT towards the lower abdomen. This observation did not apply to females with obesity (see Fig. 5, B and D). Considering SAT, especially in normal-weight females, a similar displacement of AT from the pelvis to the abdomen was observed. Females with obesity did not show any age dependency of the regional distribution of SAT, whereas males with obesity showed a similar redistribution of SAT (see Fig. 6, B to D).

## DISCUSSION

Automated MR image segmentation for the analysis of AT compartments of the body trunk using nnU-Net yields state-of-the-art performance without any manual configuration. On the basis of 30 stratified randomly selected and manually annotated samples from the GNC, the model offers robust and fast segmentation performance in terms of low SD in model evaluation metrics (Table 1) and low detection rate of uncertainty-based outliers. Moreover, an improvement of quantitative and qualitative measures compared to the literature is achieved [e.g., increase in mean DSC by 0.02 for SAT and 0.06 for VAT compared to Küstner *et al.* (*30*), respectively]. With regard to the absolute volumetric error in AT quantification, high agreement with the manual segmentation could be achieved narrowing the reported quantification error range (*29*). Only by using DL-based image processing, large data sizes can be handled in a reasonable amount of time. For example, using a trained nnUNet model for the segmentation of data from a single individual takes about 15 s compared to 3 to 4 hours of pure manual segmentation.

Qualitatively, previous studies (*30*, *34*, *35*) revealed weaknesses in the delineation of VAT leading to inaccurate AT quantification, e.g., by including intermuscular fat around the spine, vertebral bone marrow, the skeletal muscles, and parts of the pelvic cavity or by completely ignoring abdominal AT compartments besides SAT. The anatomically standardized segmentation of VAT and SAT obtained from nnU-Net overcomes these weaknesses. On the basis of unremarkable model uncertainty scores and additional manual review, the model performance directly translates to the large study population of the GNC.

Results from this work are able to confirm and extend the findings of smaller studies in terms of MR population size (*16*, *20*, *39*). First, volumetric segmentation allows assessment of regional spatial AT distribution along the craniocaudal axis leveraging the high spatial resolution of the MR data. A recent study evaluates the association of AT volumes with cardiometabolic diseases but omits the aforementioned advantages using models based on 2D projection images (*40*). Second, increased population size will allow correlations with anthropometric data, age effects, and sex differences in a fine-grained way (*9*).

In the GNC, more than 95% of the participants are Caucasians (*22*), and thus, the presented results most likely reflect this ethnicity with generalizable accuracy. However, because the relations between different AT compartments and their distribution within different ethnic groups are similar within these groups, the described methods are also applicable and can be used to describe other ethnic groups in a similar manner.

Furthermore, MRI-assessed AT compartments, especially VAT, yield deeper insight compared to basic anthropometric measures. Independent of gender, anthropometric measures (age, height, weight, and WC) explain 75% of the variation in VAT corresponding to an estimated standard error of 1.18 liters in men and 0.77 liters in women, respectively. In a study using dual-energy x-ray absorptiometry, it has been reported that approximately 90% of the variation in total fat mass is explained by age, height, weight, and ethnicity (*41*). This study now provides information about the impact of age and simple anthropometric measures on the variation of SAAT, STAT, and VAT (table S1). Together, this study does not intend to abandon any common (and cost effective) measures of abdominal obesity in favor of (expensive) MRI but to show the potential of this technique in terms of differentiation of AT compartments, their volumetric quantification and the possibility of future research of AT distribution along the craniocaudal axis—all of these aspects will probably help in characterizing the general population, taking into account the individual risk for metabolic diseases.

This study has some limitations. First, moderate IRS of the main annotator introduces noise to the training labels of the segmentation model. This noise can lead to systematic errors that are hard to detect as the model is optimized to reproduce the manual segmentations. Second, the automated detection of the region of interest can also introduce small systematic errors by missing VAT depots accumulated close to the diaphragm. Third, this study does not evaluate additional variables associated with diseases (metabolic data or laboratory parameters) and is limited to basic anthropometric data and image-based parameters. Fourth, an independent annotated testing dataset is missing and manual inspection of all segmentation results is not feasible because of the size of the population. However, the low true-positive rate (7%) of the outlier detection in combination with a manual check for anomalies (10% of the study population) sufficiently minimizes the probability of unidentified error. Fifth, the applied Dixon technique just allows a binary decision in the presence of fat mass and is not capable to detect small amounts of ectopic fat (e.g., in the liver, pancreas, or skeletal muscles), which would add important additional information on the metabolic condition of the individuals (*14*, *16*, *39*).

In conclusion, the results presented demonstrate the effectiveness of the nnU-net model to provide automated assessment of the volume and topography of AT in humans, with accuracy and precision equivalent to that of skilled human observers. This approach and the results obtained from the large population of the GNC are relevant for both epidemiological and clinical perspectives. On the basis of automated MR image analysis, meaningful epidemiological data illustrating prevalence and associated cardiometabolic disease burden of AT compartments allow to identify gender-specific and regional characteristics. By complementing existing risk prediction models with characterization of body fat distribution, improved and individualized risk estimation will be possible, as earlier identification of individuals at risk will lead to more timely and individualized prevention and treatment.

## MATERIALS AND METHODS

### German National Cohort

The GNC (NAKO Gesundheitsstudie) is a population-based, longitudinal multicentric cohort study in Germany enrolling >200,000 participants selected randomly from the population. Its main objective is to identify and to characterize risk factors for major chronic diseases (e.g., diabetes mellitus and cancer) (*22*). For a subset of approximately 30,000 participants, whole-body MRI examinations have been conducted at five imaging sites using dedicated neurologic, cardiovascular, thoracoabdominal, and musculoskeletal imaging protocols (*42*). All local on-site institutional review boards in charge of the five imaging sites approved the GNC, and written informed consent of all participants was obtained before study enrollment.

### MRI data acquisition

MRI was performed at five sites using 3-T whole-body scanners (all MAGNETOM Skyra, Siemens Healthineers, Erlangen, Germany) using a standardized acquisition protocol (*42*). Imaging of the body trunk was performed using a dedicated T1-weighted 3D VIBE two-point DIXON sequence in axial orientation with 3 mm in section thickness, 1.4 mm–by–1.4 mm in-plane voxel size, echo times of 1.23 and 2.46 ms, and a repetition time of 4.36 ms according to the GNC protocol (*42*). From this, fat- and water-selective images are automatically calculated on the scanners. For the sake of data minimization, only fat-selective images are used in the analyses. The data used in this study were obtained from the first GNC release of MRI data, which includes 11,191 participants being screened between May 2014 and December 2016.

### Segmentation model

For the automated, retrospective analysis of the MR data, a stratified (age and BMI) random sample of 30 (15 males and 15 females; demographics are provided in Table 2) manually segmented fat-selective MR images was used to train a 3D U-Net model (nnU-Net, full-resolution configuration) (*36*) to perform the segmentation of VAT and SAT. The 3D nnU-Net model was trained out of the box for 1000 epochs using fivefold cross-validation providing the mean (i.e., the output of an ensemble) of the five resulting independent models as resulting segmentation as suggested by the authors (*36*). The model-generated segmentations were evaluated by DSC and by the actual volume of the AT compartments and the percentage of the error.

### Manual segmentation

The manual labeling process was performed by a doctoral student under the supervision of two experienced medical physicists. IRS was assessed after a 2-month interruption by resegmentation from scratch of randomly chosen axial slices from the 30 originally annotated datasets.

**Fig. 7.**
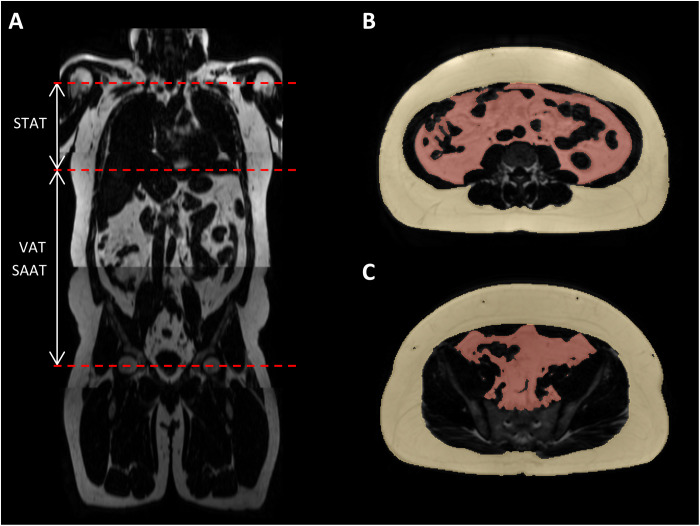
Manual segmentation. (**A**) Red dashed lines indicating the limits of the ranges of manual segmentation (femoral heads to cardiac apex for VAT and SAAT and cardiac apex to humeral heads for STAT, respectively) and (**B** and **C**) axial examples of manual segmentations of SAT (yellow) and VAT (red).

To assess VAT, defined as AT inside the abdominal cavity including retroperitoneal structures such as the kidneys, pancreas, or duodenum, in a standardized manner, AT accumulated around the heart is excluded. The manual segmentation was performed from the middle of the femoral heads to the cardiac apex, since the thoracic diaphragm cannot be detected on the MR images. SAT was segmented ranging from the middle of the femoral heads to the middle of the humeral heads (see red dashed lines in Fig. 7 indicating the different levels). By design, nnU-Net implicitly recognizes these inferior and superior boundaries with no need for an explicit adaption of the model to the region of interest.

### Uncertainty-based outlier detection

Because of the large population size of the cohort, an automated measure for outlier detection in the segmentation based on mean pairwise DSC of the cross-validated training folds to estimate the model’s uncertainty (*43*) was used. Participants are classified as outliers if the model uncertainty is three interquartile ranges below the first quartile of all participants. Detected outliers were reviewed manually by three different scientists. In addition, random datasets up to 10% of the study cohort were manually checked for anomalies.

### Fat quantification and spatial distribution

For the analysis of the associations of AT depots and anthropometric data, SAT was differentiated in SAAT and STAT using the cardiac apex as the boundary. The regional spatial distribution of the AT compartments was described by considering the percentage of total AT of the trunk along the craniocaudal axis.

### Anthropometric data

Body height and weight were assessed using standardized measuring instruments across the study centers (all Stadiometer 274 for height and medical Body Composition Analyzer 515 for weight, both seca GmBH, Hamburg, Germany). WC was measured at the midpoint between the iliac crest and the lowest rib. The study participants should come to the measurements with an empty bladder, should not have been physically active in the last hour, and should not lie down 10 min before the measurements (*44*). For further analyses, normal weight was defined as BMI ≤ 25 kg/m^2^, whereas obesity was defined as BMI ≥ 30 kg/m^2^.

### Statistical analysis

Data are reported as means ± SD unless stated otherwise. Bland-Altman plots are were used to visualize the agreement between manual and automated AT quantification. IRS was measured as DSC using true-positive, false-positive, and false-negative annotated image pixels. Model performance was evaluated using class-wise metrics of the confusion matrix, e.g., DSC or precision. The association of AT compartments and anthropometric (gender, age, and BMI) data was assessed using linear regression and Pearson’s correlation coefficient. Two-sample Welch’s *t* test was used for the determination of gender-related differences. The explained variation in MRI-assessed AT compartments by anthropometric measures is modeled using multiple linear regression models. SEM was used in the visualization of regional AT distribution. *P* < 0.05 was considered statistically significant in this study. All statistical analyses were performed in Python 3.8 using SciPy 1.5.4 and R version 4.2.0.

## Notes

View/request a protocol for this paper from *Bio-protocol*.
