# Supplementary material for: Analysis of volume and topography of adipose tissue in the trunk: Results of MRI of 11,141 participants in the German National Cohort
Source: Sci Adv. 2023 May 12;9(19):eadd0433. doi: 10.1126/sciadv.add0433 (PMC10181183; doi:10.1126/sciadv.add0433)
Supplement: Supplementary file 1 — Table S1 [file sciadv.add0433_sm.pdf]

Science Advances
AAAS

Supplementary Materials for

Analysis of volume and topography of adipose tissue in the trunk: Results of MRI of 11,141 participants in the German National Cohort

Tobias Haueise *et al.*

Corresponding author: Juergen Machann, juergen.machann@med.uni-tuebingen.de

Sci. Adv. **9**, eadd0433 (2023)
DOI: 10.1126/sciadv.add0433

This PDF file includes:

Table S1

Table S1. Multiple Linear Regression Models. Prediction of the variation in MRI assessed adipose tissue compartments using data routinely collected in clinical practice.

Dependent variables	Age†	Height†	Weight†	WC†	R^2	SEE (l)
SAAT						
Male (n = 5697*)						
Model 1	-0.057	-0.292	0.967	-	0.79	1.39
Model 2	-0.137	-0.185	0.594	0.393	0.81	1.33
Female (n = 5420*)						
Model 1	-0.008	-0.248	0.968	-	0.89	1.29
Model 2	-0.045	-0.201	0.758	0.233	0.90	1.24
STAT						
Male (n = 5697)						
Model 1	0.108	-0.258	0.876	-	0.69	0.65
Model 2	0.018	-0.138	0.454	0.444	0.72	0.62
Female (n = 5420)						
Model 1	0.130	-0.251	0.866	-	0.77	0.84
Model 2	0.070	-0.173	0.522	0.383	0.80	0.79
VAT						
Male (n = 5697)						
Model 1	0.301	-0.228	0.783	-	0.68	1.34
Model 2	0.152	-0.028	0.085	0.734	0.75	1.18
Female (n = 5420)						
Model 1	0.290	-0.189	0.753	-	0.70	0.85
Model 2	0.200	-0.073	0.236	0.576	0.75	0.77

SAAT subcutaneous abdominal adipose tissue, *SEE* standard error of estimate, *STAT* subcutaneous thoracic adipose tissue, *VAT* visceral adipose tissue, *WC* waist circumference

*Analysis included all subjects with WC data available

†Standardized beta coefficients
